# Associations between complex post-traumatic stress disorder and intimate relationship quality: the parallel mediating roles of mentalization and playfulness

**DOI:** 10.3389/fpsyg.2026.1779202

**Published:** 2026-05-01

**Authors:** Shu-Xin Tan, Hang Li, Chengjing Chu, Jun-Wen Tan

**Affiliations:** 1 School of Humanities and Management, Guangdong Medical University, Dongguan, China; 2 Key Laboratory for Quality of Life and Psychological Assessment and Intervention, Guangdong Medical University, Dongguan, China

**Keywords:** complex trauma, CPTSD, intimate relationship quality, mentalization, playfulness

## Abstract

**Background:**

The association between Complex Post-Traumatic Stress Disorder (CPTSD) and intimate relationship quality remains underexplored, particularly regarding protective factors that may buffer against trauma-related impairments.

**Methods:**

A total of 3,250 participants completed self-report surveys assessing CPTSD symptoms, intimate relationship quality, mentalization, and playfulness, with 12.8% (*N* = 416) meeting criteria for probable CPTSD. A dynamic network heatmap and Mantel-based network analysis revealed distinct connectivity patterns. Structural equation modeling (SEM) was used to examine the relationships among the variables.

**Results:**

Our study indicates that CPTSD symptoms formed a negatively correlated network with relationship quality indicators. Structural equation modeling further showed that CPTSD was negatively associated with relationship quality, mentalization, and playfulness; while mentalization and playfulness positively associated with relationship quality. Mentalization and playfulness served as parallel mediators in the association between CPTSD and relationship quality.

**Conclusions:**

The present study is the first to reveal the dual mediating roles of mentalization and playfulness in the association between CPTSD and intimate relationship quality. Our findings suggest that enhancing mentalization and playfulness may serve as effective intervention strategies for improving relationship quality among individuals exhibiting symptoms of CPTSD.

## 1 Introduction

Complex Post-Traumatic Stress Disorder (CPTSD) is often associated with prolonged exposure to chronic interpersonal trauma (e.g., childhood abuse or neglect) and encompasses not only the core symptoms of Post-Traumatic Stress Disorder (PTSD) but also “Disturbances in Self-Organization” (DSO), including affective dysregulation, interpersonal difficulties, and negative self-concept (Cloitre et al., 2019; World Health Organization, 2018). These features pose significant challenges to the formation and quality of intimate relationships. Consistent with prior research, empirical evidence has linked CPTSD to heightened negative self-concept, difficulties in emotion regulation, and attachment insecurity, in particular, attachment anxiety, which is characterized by fear of rejection and distress when partners are unresponsive (Karatzias et al., 2018, 2022). However, the psychological mechanisms underlying these relationship difficulties remain insufficiently understood, limiting progress in developing targeted interventions.

Intimate relationships, which are fundamental to human wellbeing, are pivotal to positive emotions, psychological stability, and life satisfaction (Love and Holder, 2016; Baumeister and Leary, 2017; Csikszentmihalyi et al., 2014). High-quality relationships are further linked to greater subjective wellbeing, reduced stress, and lower depression levels (Tan et al., 2023; Braithwaite and Holt-Lunstad, 2017; Proulx et al., 2007). As a secure base for emotional expression, validation, and the development of resilience (Mikulincer and Shaver, 2019), intimate partnerships may be particularly vulnerable to disruptions associated with CPTSD.

Individuals with CPTSD frequently grapple with self-perceptions of guilt, helplessness, shame, and a pervasive fear of intimacy, alongside emotional numbness or inhibition (Dorahy et al., 2013; Maercker et al., 2013). These experiences contribute to significant relational challenges, including the avoidance of emotional closeness and heightened conflict, ultimately affecting relational satisfaction and stability (Mikulincer and Shaver, 2010; Cloitre et al., 2019). It is crucial to examine how CPTSD affects intimate relationships, as its symptoms can create substantial barriers to emotional connection and communication, necessitating tailored therapeutic approaches to address these challenges (Bachem et al., 2021; Yang et al., 2023). Moreover, previous research has suggested that reducing individual psychopathological symptoms does not necessarily improve relationship problems; in contrast, addressing relationship distress and enhancing relational functioning can yield benefits for both relational quality and mental health (Whisman and Baucom, 2012). However, the precise mechanisms through which CPTSD impacts the quality of intimate relationships remain underexplored. The present study aims to clarify the association between CPTSD and romantic relationship quality and to investigate resilience-related factors, mentalization and playfulness.

### 1.1 The role of mentalization

Mentalization refers to the capacity to understand and interpret one's own and others' behaviors in terms of underlying psychological states, such as beliefs, desires, emotions, and intentions (Luyten et al., 2020). This capacity is essential for maintaining trust, emotional understanding, and effective communication in close relationships (Fonagy et al., 2015). Traumatic experiences—such as childhood abuse, neglect, or exposure to interpersonal violence—can disrupt relational functioning by fostering insecure attachment, emotional dysregulation, and interpersonal conflict (Mikulincer and Shaver, 2012; Mozafari and Xu, 2025; Zurbriggen et al., 2012). These experiences also impair mentalization capacities, reducing individuals' ability to accurately interpret others' intentions and thereby increasing vulnerability to interpersonal misunderstandings and relationship difficulties, as described in attachment and mentalization frameworks (Fonagy and Bateman, 2006).

Secure attachment in early life provides a developmental foundation for mentalization, which subsequently supports emotional understanding and regulation within close relationships (Fonagy and Luyten, 2009; Midgley and Vrouva, 2013). In the context of trauma, individuals with higher levels of mentalization appear better equipped to navigate relational difficulties. For example, (Berthelot et al. 2015) found that mothers with stronger mentalizing capacities were more effective in buffering the negative effects of trauma on family and intimate dynamics. In trauma-affected populations, such as individuals with CPTSD, mentalization has been posited as a protective mechanism that mitigates interpersonal dysfunction (Shayegh et al., 2025). Higher mentalization enables individuals to better understand their partner's emotional perspectives during conflict, thereby reducing misinterpretation and emotional reactivity (Fonagy and Target, 2006; Górska and Marszał, 2014; Matin et al., 2025). It also promotes empathy and emotional attunement, both of which are crucial for relationship satisfaction, particularly in trauma-impacted partnerships characterized by mistrust and misunderstanding (Jurist and Meehan, 2009). Research further suggests that couples who demonstrate greater mentalizing in their interactions report increased trust, intimacy, and overall relationship quality (Gottman and Levenson, 2000; Nyberg and Hertzmann, 2018). In the context of post-traumatic psychotherapy, enhanced mentalization can facilitate the repair of damaged intimate relationships (Allen et al., 2008; Bateman and Fonagy, 2016; Bleiberg et al., 2023). Mentalization-based interventions have shown promise in improving relational outcomes; for instance, (McCann 2022) reported enhanced relationship satisfaction and stability among high-conflict couples following mentalization-based couples therapy.

### 1.2 The role of playfulness

Playfulness refers to an individual's tendency to frame situations in a light-hearted, humorous, and flexible manner (Masek, 2025; Proyer, 2014a,b). As a positive psychological disposition, it facilitates emotional expression, interpersonal bonding, and adaptive functioning. In romantic relationships, playfulness has been associated with greater emotional intimacy, constructive conflict resolution, and higher relationship satisfaction (Proyer et al., 2019; de Moraes et al., 2021). It typically manifests through shared humor, spontaneous activities, and playful banter-behaviors that foster emotional safety and closeness between partners.

Accumulating evidence indicates that playfulness may serve as a protective factor in relationships experiencing psychological distress. Playfulness may both serve as an outlet for stress and negative emotions and may play an important role in sustaining mental health (Lubbers et al., 2023; Shen and Masek, 2024). However, engaging in playful behavior can be particularly challenging for individuals with CPTSD, a condition characterized by emotional dysregulation and persistent relational disturbances (Cloitre et al., 2009; Hyland et al., 2023; Van der Kolk, 2002). Emotional numbing and difficulties with trust may restrict one's capacity for spontaneity and openness, thus limiting opportunities for light-hearted engagement within intimate relationships (Butler et al., 2022; Turbessi, 2023).

Despite these challenges, playfulness may nonetheless support trauma survivors in rebuilding emotional connections and alleviating relational strain. First, it appears to operate as a coping and resilience mechanism in the face of stress and adversity. During the COVID-19 pandemic, for instance, higher adult playfulness was associated with greater adaptive coping, stronger perceived self-efficacy, and lower helplessness (Clifford et al., 2024). Similarly, in a war-affected sample, network analyses highlighted that adult playfulness (particularly lighthearted playfulness) was closely linked to resilience and lower clinical symptom severity (Rubinstein et al., 2025). Second, playfulness may facilitate positive interpersonal dynamics by fostering intimacy, mutual enjoyment, and adaptive communication within close relationships (Brauer et al., 2023a). Shared laughter between romantic partners, for instance, has been found to correlate positively with relationship quality, intimacy, and perceived support (Kurtz and Algoe, 2015). Moreover, interventions designed to enhance playfulness have demonstrated improvements in wellbeing and reductions in depressive symptoms (Proyer et al., 2021). Collectively, these studies suggest that playfulness functions both as a positive psychological resource and as a valuable interpersonal asset, potentially helping individuals with CPTSD enhance their emotional adaptability and improve their interpersonal relationships.

### 1.3 The present study

**Figure 1:**
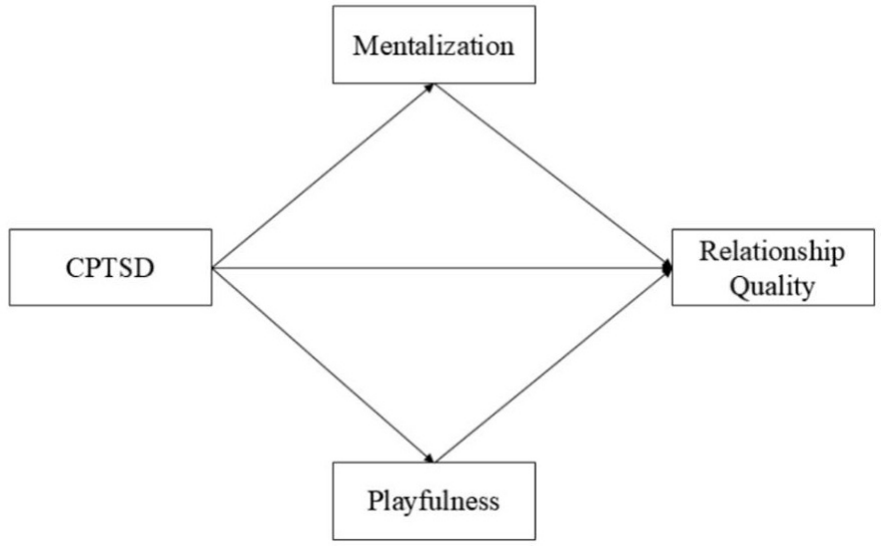
The hypothesized model of the associations between CPTSD and intimate relationship quality.

The present study aims to examine the roles of mentalization and playfulness in the relation between CPTSD and the quality of intimate relationships. Grounded in prior research, this study proposes the following hypotheses: (1) Symptoms of CPTSD are negatively associated with intimate relationship quality, levels of mentalization, and playfulness; (2) Mentalization and playfulness are positively associated with intimate relationship quality; (3) Mentalization and playfulness function as parallel mediators in the association between CPTSD symptoms and relationship quality. The conceptual framework is shown in Figure 1.

## 2 Materials and methods

### 2.1 Participants and procedures

This study recruited 4,427 participants from China using a convenience sampling method that combined online questionnaire surveys. The survey link was disseminated via general public online platforms, and individuals voluntarily chose to participate. Participants were drawn from the general community and did not represent clinical or treatment-seeking adults. Eligible participants were adults currently engaged in intimate romantic relationships, including dating or marital relationships.

This study involved an anonymous online survey in which no names or personally identifying information were required. Participants were presented with an informed consent statement at the beginning of the survey, which outlined the purpose of the study, their rights to confidentiality, and their ability to withdraw at any time without consequence. Electronic informed consent was obtained from all participants prior to the commencement of the survey, and their participation was entirely voluntary. Participants then completed a comprehensive set of questionnaires, which included demographic variables, the International Trauma Questionnaire–Chinese Version, the Short Measure of Adult Playfulness–Chinese Version, the Perceived Relationship Quality Components Inventory, and the Mentalization Scale.

Participants were excluded according to three *a priori* criteria: (1) being single, which did not meet the study's eligibility requirement of current romantic involvement (*n* = 470); (2) survey completion time less than 5 min, indicating inattentive responding; (3) excessive repetitive or invariant responding, characterized by long strings of identical responses (Meade and Craig, 2012). All exclusions were based on *a priori* eligibility and data quality standards rather than systematic or differential attrition. After this screening, a total of 3,250 valid cases were collected, of the valid participants, 46.9 % were female (*N* = 1,523), and 53.1 % were male (*N* = 1,727). Based on the self-reported ITQ cut-off criteria, 12.8% of participants in valid sample met the criteria for probable CPTSD, resulting in a final sample of 416 participants for further analyses. This study was conducted in accordance with the Declaration of Helsinki and was approved by the Ethics Review Committee of Guangdong Medical University (YJYS2023293). Clinical trial number: not applicable.

### 2.2 Measures

#### 2.2.1 International trauma questionnaire (ITQ)

The International Trauma Questionnaire (ITQ; Cloitre et al., 2018) is an 18-item self-report questionnaire was used in this study to access CPTSD symptoms. The ITQ contains three symptoms that evaluate PTSD: Re-experiencing in the here and now (Re), Avoidance (Av), and Sense of current threat (Th). Each symptom is assessed with two items, and three additional items evaluate functional impairment related to PTSD symptoms. The DSO also contains three symptoms: Affective dysregulation (AD), Negative self-concept (NSC), and Disturbances in relationships (DR). Each symptom is assessed with two items, and functional impairment related to DSO is assessed with three items.

The criteria of PTSD or DSO was met when at least one of the two items of each associated symptom, scored 2 (moderately) or higher, and functional impairment (at least one of the three items scored 2 or higher) was observed simultaneously. Participants were classified as having probable CPTSD when they met the criteria for both PTSD and DSO.

All ITQ items were answered on a five-point Likert scale ranging from 0 (Not at all) to 4 (Extremely). Participants rated how “bothered” they were by their symptoms in the past month (0 = not at all to 4 = extremely). The reliability and validity of the ITQ Chinese version have been clinically validated in Chinese samples (Ho et al., 2019; Tian et al., 2020). In the present study, the Cronbach's alpha internal consistency reliability coefficients for the ITQ were 0.84 for PTSD and 0.85 for DSO.

#### 2.2.2 Perceived relationship quality components (PRQC) inventory

The Perceived Relationship Quality Component (PRQC) inventory was employed to assess participants' relationship quality (Fletcher et al., 2000). The PRQC includes 18 items (e.g., “How satisfied are you with your relationship?”), measures six dimensions of relationship quality: relationship satisfaction, commitment, trust, intimacy, passion, and love, and each dimension was examined through three items, using a seven-point Likert-type scale ranging from 1 (not at all) to 7 (extremely). Higher ratings indicate better relationship quality. The reliability and validity of the PRQC Chinese version have been validated in Chinese samples (Zhou et al., 2017). In this study, the Cronbach's alpha for the PRQC is 0.94.

#### 2.2.3 The short measure of adult playfulness (SMAP)

The Short Measure of Adult Playfulness (SMAP; Proyer, 2012) is a brief psychometric tool designed to assess playfulness in adults. Playfulness, as measured by SMAP, is conceptualized as an individual's tendency to engage in activities with a light-hearted, spontaneous, and flexible manner. The scale comprises five items rated on a seven-point Likert scale ranging from 1 (“strongly disagree”) to 7 (“strongly agree”). Although a revised structural model of playfulness was later proposed (Proyer, 2017), the SMAP was chosen in the present study for its brevity, established reliability, and demonstrated validity in Chinese samples (Pang and Proyer, 2018), ensuring both cultural appropriateness and comparability with prior research. The Chinese version of the SMAP has been validated in measuring adult playfulness in Chinese samples (Pang and Proyer, 2018). The internal consistency coefficient for the SMAP in the present study was 0.88.

#### 2.2.4 The mentalization scale (MentS)

The Mentalization Scale (MentS; Dimitrijević et al., 2018) was used to assess for indications of mentalization. The MentS consists of 28 items evaluates mentalization as a general construct, as well as three potential dimensions: Self-Related Mentalization (MentS-S), Other-related Mentalization (MentS-O), and Motivation to Mentalize (MentS-M). Each statement is answered on a five-point Likert-type scale (ranging from 1 = Completely incorrect to 5 = Completely correct). A higher score indicates a higher degree of mentalization. The reliability and validity of the MentS Chinese version have been validated in Chinese samples (Huang and Hou, 2023). In the current sample, the Cronbach's alpha coefficient for this scale is 0.95.

### 2.3 Data analysis

Statistical analysis was performed using IBM SPSS Statistics 27.0 (IBM Corp., Armonk, NY, USA), with statistical significance set at *p* < 0.05. Demographic characteristics and descriptive statistics as well as bivariate associations were examined, including frequencies, percentages, means, and standard deviations. To test the hypothesized mediation model, the structural equation modeling (SEM) was performed by IBM SPSS Amos 28.0 (IBM Corp., Armonk, NY, USA) with the maximum likelihood estimation method.

To visualize and explore the structural configuration of symptom–relationship associations, a dynamic network heatmap and network visualization were generated using https://www.omicshare.com (Mu et al., 2024). Pairwise correlations among CPTSD symptom clusters and intimate relationship quality indicators were computed, and the resulting correlation matrix was visualized as a heatmap, while the network structure was visualized based on the strength and direction of associations (Knefel et al., 2019; Schiess-Jokanovic et al., 2022).

The model fit was assessed using multiple indices, including the likelihood ratio statistic, CFI, TLI, RMSEA, and GFI (Hu and Bentler, 1999). To test the indirect effects, 5,000-sample bias-corrected bootstrap confidence intervals were calculated. The mediating effect was considered statistically significant at the 0.05 level, if the 95% confidence interval did not include zero.

## 3 Results

### 3.1 Sample characteristics

According to the self-reported ITQ cut-off criteria, 12.8% of participants in this sample met the criteria for probable CPTSD (i.e., meeting both PTSD and DSO criteria). In addition, 15.8% (*n* = 512) met PTSD-only criteria, and 14.9% (*n* = 483) met DSO-only criteria. The final probable CPTSD sample consisted of 416 participants (57.5% female, *M* = 25.09 years, *SD* = 4.72; 42.5% male, *M* = 25.69 years, *SD* = 4.66). Table 1 presents the detailed demographic of the sample.

**Table 1:** Probable CPTSD sample demographics.

Variable	*N* (%)
Gender	
Female	239 (57.5)
Male	177 (42.5)
Duration of relationship	
Within half a year	48 (11.5)
6 months to 1 year	146 (35.1)
1–2 years	127 (30.5)
2–3 years	29 (7.0)
More than 3 years	66 (15.9)
Education	
Junior high school completion	4 (1.0)
High school completion	32 (7.7)
College student	194 (46.6)
College completion	117 (28.1)
Post-graduate student	59 (14.2)
Graduate degree completion	10 (2.4)
Rural areas	118 (28.4)
Long-distance relationship	112 (26.9)
Married	134 (32.2)

N = 416.

### 3.2 Bivariate correlations among study variables

Before testing the model, the correlations among the variables were examined, as well as the mean and standard deviation of all studied variables, presented in Table 2. The dimensions of relationship quality were significantly negatively correlated with the two latent dimensions of CPTSD, which is PTSD and DSO. The PTSD and DSO were negatively correlated with mentalization and playfulness. Playfulness and mentalization were significantly positively correlated with dimensions of relationship quality. These results support further investigation of relationships using path models.

**Table 2:** Descriptive statistics and correlations between study variables.

Variables	1	2	3	4	5	6	7	8	9	10
1. Relationship satisfaction	1									
2. Commitment	0.68^**^	1								
3. Intimacy	0.76^**^	0.71^**^	1							
4. Trust	0.67^**^	0.73^**^	0.79^**^	1						
5. Passion	0.67^**^	0.68^**^	0.73^**^	0.71^**^	1					
6. Love	0.57^**^	0.63^**^	0.67^**^	0.73^**^	0.68^**^	1				
7. Mentalization	0.49^**^	0.48^**^	0.51^**^	0.47^**^	0.47^**^	0.41^**^	1			
8. Playfulness	0.50^**^	0.40^**^	0.45^**^	0.40^**^	0.45^**^	0.32^**^	0.53^**^	1		
9. PTSD	−0.56^**^	−0.47^**^	−0.53^**^	−0.49^**^	−0.48^**^	−0.39^**^	−0.59^**^	−0.53^**^	1	
10. DSO	−0.38^**^	−0.41^**^	−0.42^**^	−0.45^**^	−0.39^**^	−0.33^**^	−0.58^**^	−0.45^**^	−0.75^**^	1
*M*	8.59	8.40	8.60	8.49	8.72	8.21	67.30	15.30	15.33	15.26
*SD*	2.65	2.56	2.64	2.64	2.77	2.81	15.44	4.91	3.18	3.34

PTSD, post-traumatic stress disorder; DSO, disturbances in self—organization.

^**^p < 0.01.

### 3.3 Network analysis of CPTSD symptoms and the quality of intimate relationships

**Figure 2:**
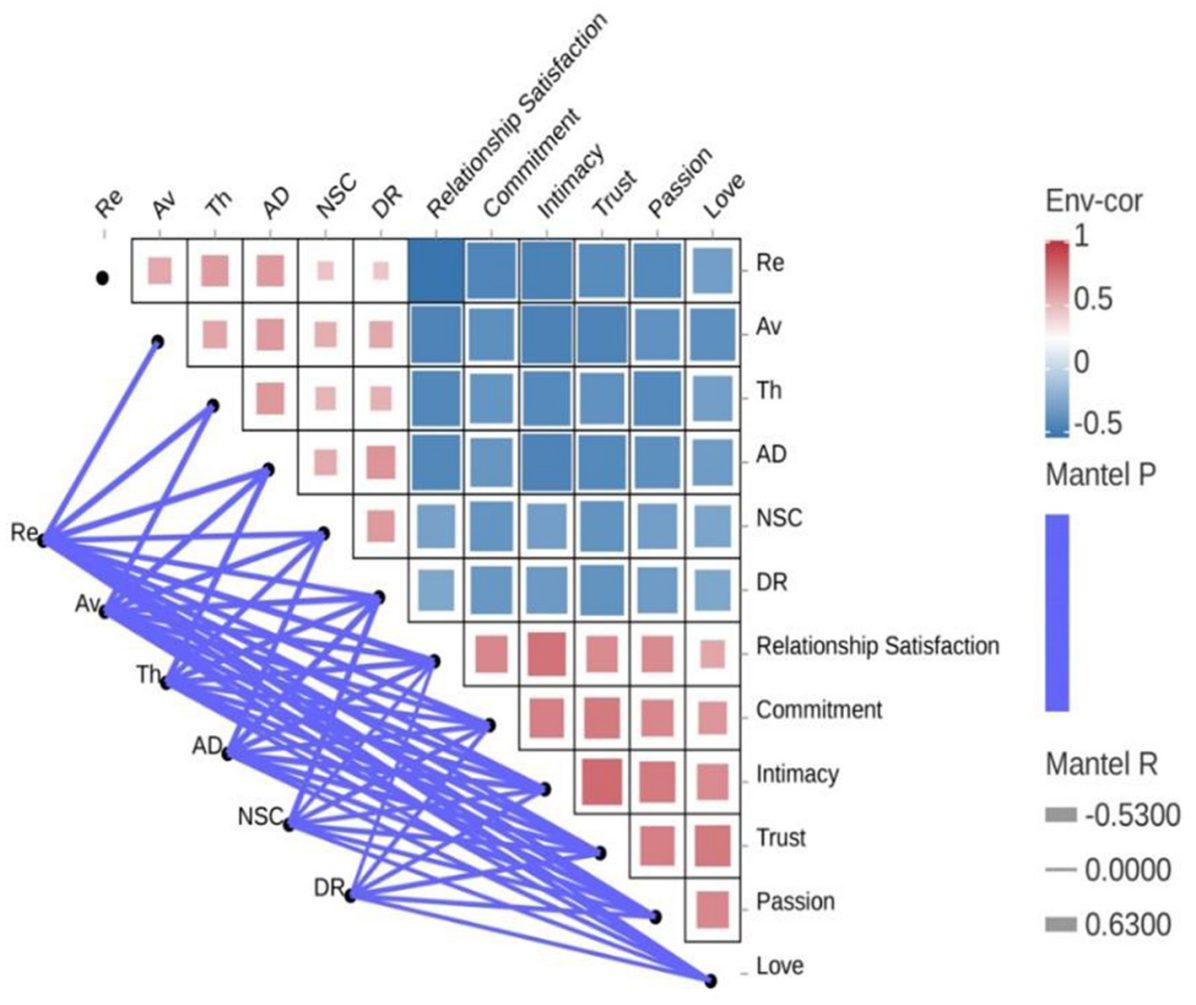
Network topology and heatmap visualization of CPTSD symptom clusters and their differential associations with intimate relationship quality. Blue squares indicate negative correlations, while red squares indicate positive correlations. The intensity of the color reflects the strength of the correlation, with the color scale capped at −0.50 for visualization clarity. Re, re-experiencing in the here and now; Av, avoidance; Th, sense of current threat; AD, affective dysregulation; NSC, negative self-concept; DR, disturbances in relationships.

Figure 2 presents a network heatmap visualization illustrating the Pearson correlations between CPTSD symptom clusters and intimacy-related domains, coupled with Mantel test validation of the overall association pattern. Overall, the network exhibited a clear modular structure, separating CPTSD symptom clusters (Re, Av, Th, AD, NSC, DR) from intimacy-related domains (relationship satisfaction, commitment, intimacy, trust, passion, and love). For visualization clarity, the color scale was capped at −0.50.

Cross-module comparisons (i.e., between CPTSD symptom clusters and intimacy-related domains) were characterized by uniformly negative correlations (*r* = −0.26 to −0.53), as indicated by the blue heatmap cells. The strongest negative cross-module associations emerged for Re and AD with core relational functioning indicators, particularly relationship satisfaction, intimacy, and trust. In contrast, within-module associations (i.e., correlations among variables belonging to the same domain) were consistently positive, as indicated by the red heatmap cells. This pattern was observed within both the CPTSD symptom clusters and the intimacy-related domains; however, correlations were stronger among the intimacy-related variables (*r* = 0.57–0.79), indicating a higher degree of internal coherence within the relational functioning subsystem.

To examine the global association between CPTSD symptom structure and intimate relationship functioning, a Mantel test was conducted. The test revealed a significant negative environmental correlation (Mantel *R* = −0.53, *p* < 0.001), indicating that the overall configuration of CPTSD symptoms was systematically and inversely aligned with the configuration of relational functioning domains. This result supports the network-level inference that increasing CPTSD symptom severity corresponds to widespread impairment across intimacy-related domains, rather than isolated pairwise effects.

Collectively, these findings indicate that CPTSD symptoms are systematically and negatively correlations with intimate relationship quality.

### 3.4 Structural equation modeling

**Figure 3:**
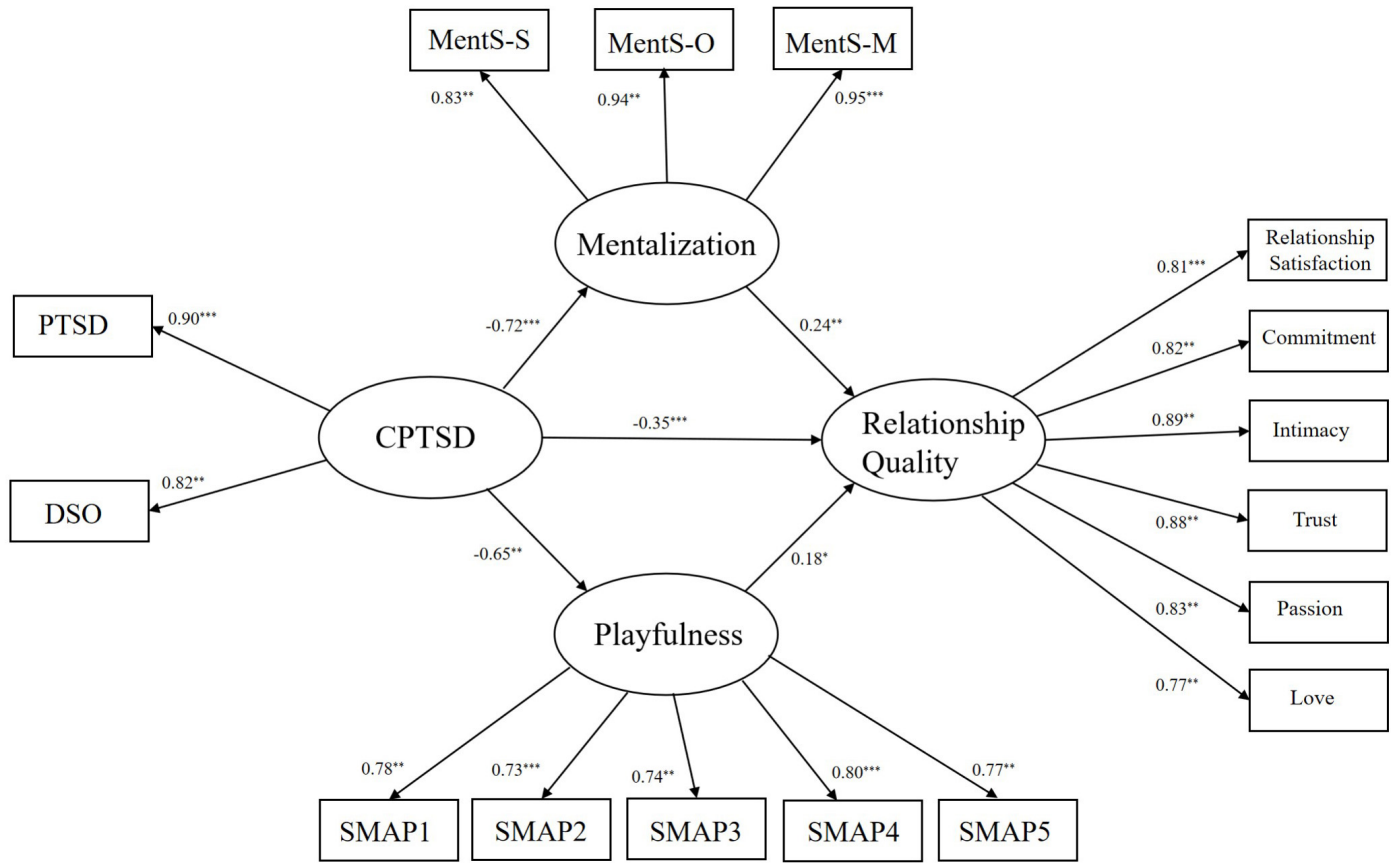
Structural equation model of CPTSD, mentalization, playfulness and relationship quality. CPTSD, complex post-traumatic stress disorder; PTSD, post-traumatic stress disorder; DSO, disturbances in self-organization; SMAP, the short measure of adult playfulness; SMAP1, SMAP item1; SMAP2, SMAP item2; SMAP3, SMAP item3; SMAP4, SMAP item4; SMAP5, SMAP item5; MentS-S, self-related metallization; MentS-O, other related mentalization; MentS-M, motivation to mentalize. *p < 0.05, **p < 0.01, ***p < 0.001.

As presented in Figure 3, structural equation model was conducted to test hypothesized model, analysis of Mentalization and playfulness in the link between CPTSD and relationship quality. This model fit the data well, as indicated by the fit indices: χ^2^/df = 2.59, *p* < 0.001, root mean square error of approximation (RMSEA) = 0.06; Tucker–Lewis index (TLI) = 0.96; Comparative Fit Index (CFI) = 0.97; Goodness-of-fit index (GFI) = 0.93. The Bootstrap method was used for testing; sampling was repeated 5,000 times and 95% was set the confidence interval.

As shown in Table 3 and Figure 3, all direct and indirect pathways in the parallel mediation model were statistically significant. Notably, the bootstrap 95% confidence intervals for both mediators excluded zero, confirming the presence of parallel mediation effects. Specifically, CPTSD was significantly negatively associated with mentalization [β = −0.72, *p* < 0.001, 95% CI (−0.79, −0.64)] and with playfulness [β = −0.65, *p* < 0.01, 95% CI (−0.73, −0.54)] CPTSD was also negatively associated with relationship quality [β = −0.35, *p* < 0.001, 95% CI (−0.52, −0.17)]. In contrast, mentalization was positively associated with relationship quality [β = 0.24, *p* < 0.01, 95% CI (0.09, 0.39)], and playfulness was also positively associated with relationship quality [β = 0.18, *p* < 0.05, 95% CI (0.03, 0.34)]. Collectively, these findings were consistent with the hypothesized mediation model involving mentalization and playfulness.

**Table 3:** Standardized direct and indirect effects, total effects, and mediated proportions for the association between CPTSD and relationship quality.

Path/effect	β	95% CI	*p*-Value
Direct paths			
CPTSD → mentalization	−0.72	[−0.79, −0.64]	< 0.001
CPTSD → playfulness	−0.65	[−0.73, −0.54]	0.001
Mentalization → relationship_quality	0.24	[0.09, 0.39]	0.002
Playfulness → relationship_quality	0.18	[0.03, 0.34]	0.015
CPTSD → relationship_quality	−0.35	[−0.52, −0.17]	< 0.001
Indirect effects			
Indirect effects1 (via mentalization)	−0.14	[−0.23, −0.05]	0.001
Indirect effects2 (via playfulness)	−0.09	[−0.20, −0.02]	0.014
Total indirect effects	−0.23	[−0.36, −0.12]	< 0.001
Total effects	−0.50	[−0.61, −0.41]	< 0.001
Mediated proportions			
r1 (Indirect1/total)	0.27	[0.10, 0.47]	0.001
r2 (Indirect2/total)	0.18	[0.04, 0.38]	0.014
Difference (indirect1—indirect2)	−0.05	[−0.17, 0.08]	0.474

The total indirect effects were significant [β = −0.23, *p* < 0.001, 95% CI (−0.36, −0.12)]. Furthermore, the indirect pathway from CPTSD to relationship quality via mentalization was significant [β = −0.14, *p* < 0.01, 95% CI (−0.23, −0.05)], as was the indirect pathway from CPTSD to relationship quality via playfulness [β = −0.09, *p* < 0.05, 95% CI (−0.20, −0.02)].The relative contribution of each indirect pathway was quantified using the proportion-mediated effect (PM; Preacher and Hayes, 2008). The indirect effect via mentalization accounted for 27% of the total effect [PM = 0.27, *p* < 0.01, 95% CI (0.10, 0.47)], while the indirect effect via playfulness accounted for 18% [PM = 0.18, *p* < 0.05, 95% CI (0.04, 0.38)]. In contrast, a comparative analysis of the two mediating effects revealed that there was no statistically significant difference between the two indirect paths [β= −0.05, *p* > 0.05, 95% CI (−0.17, 0.08)].

The analytical findings provide empirical support for the parallel mediation model, with both mentalization and playfulness simultaneously mediating the relationship between CPTSD and relationship quality.

## 4 Discussion

This study examined parallel mediation pathways of mentalization and playfulness in the CPTSD-relationship quality association, with important implications for future research and clinical practice.

Results revealed a significant negative association between CPTSD symptoms and intimate relationship quality, consistent with previous findings linking complex trauma to diminished interpersonal functioning (Bachem et al., 2021; Stadtmann et al., 2018a,b; Dorahy et al., 2013). Extending prior research documenting social withdrawal and impaired intimacy among trauma-exposed individuals (Dorahy, 2010), the current findings specify that higher CPTSD symptomatology was associated with reduced satisfaction, commitment, trust, intimacy, passion, and love. Notably, both mentalization and playfulness emerged as significant parallel mediators, with no statistically significant difference between the two indirect pathways. This suggests that cognitive-affective and behavioral-interpersonal processes operate as distinct yet complementary routes through which CPTSD is associated with relationship quality.

### 4.1 Mediating effects of the mentalization to CPTSD and intimate relationships

Results of the current study revealed that mentalization, defined as the capacity to understand and interpret one's own and others' mental states (Luyten and Fonagy, 2015; Luyten et al., 2020), plays a significant mediating role in the association between CPTSD symptomatology and the quality of intimate relationships. This finding provides insight into the psychological mechanisms that may underlie the association between CPTSD symptoms and relational functioning. Mentalization is widely regarded as a core aspect of interpersonal competence, enabling individuals to recognize and make sense of internal mental experiences such as intentions, feelings, and beliefs. In intimate relationships, higher mentalizing capacity has been associated with greater relationship satisfaction and emotional connectedness (MacIntosh et al., 2019).

In line with previous research, the current findings indicate that higher CPTSD symptoms is associated with lower mentalizing capacity, which may reflect difficulties in accurately perceiving partners' emotional states, needs, and behaviors. Reflective functioning, a core component of mentalization, has been associated with attachment patterns and relationship satisfaction, indicating its potential importance for interpersonal functioning (Ghossain, 2014; Kriplani, 2020). Consistent with prior research, the present findings indicate that lower mentalization capacity is related to lower levels of intimate relationship quality.

Moreover, these results are consistent with studies positioning mentalization as a resilience-related resource in romantic relationships, particularly among individuals with early interpersonal trauma. For instance, (Lassri and Gewirtz-Meydan 2025) found that stronger mentalizing abilities were linked to higher relationship satisfaction despite childhood adversity, underscoring the role of mentalization in mitigating the relational impacts of trauma. Similarly, (Shayegh et al. 2023) demonstrated that mentalization plays a mediating role between childhood trauma and attachment difficulties. These findings align with this body of work, highlighting the potential of mentalization to be associated with fewer mentalization is particularly relevant for understanding how trauma-related symptoms affect intimate relationships negative relational patterns often associated with CPTSD. Given that CPTSD, unlike PTSD, is characterized by chronic relational disturbances (Karatzias et al., 2018; Li et al., 2025), mentalization is particularly relevant for understanding how trauma-related symptoms relate to intimate relationships. Methodologically, this study advances the field by conceptualizing mentalization not merely as a relational skill but as a mediating psychological process in the association between CPTSD symptoms to interpersonal functioning, consistent with theoretical models highlighting mentalization as a key mechanism in trauma-related relational difficulties (Allen et al., 2008; Fonagy and Luyten, 2016; Terradas et al., 2021).

Importantly, the current findings refine and advance attachment-based theoretical frameworks by demonstrating that diminished mentalization mediates the association between CPTSD symptoms and poorer relationship quality. This is consistent with perspectives linking attachment-related processes to social cognition and mentalization (Fonagy and Bateman, 2006; Fonagy et al., 2023; Sikand et al., 2025). Furthermore, these findings underscore the necessity of integrated interventions targeting both cognitive-affective regulation and relational dynamics in CPTSD treatment (Stadtmann et al., 2018a,b).

### 4.2 Mediating effects of the playfulness to CPTSD and intimate relationships

Playfulness can be defined as the ability of an individual to frame or redefine everyday situations in a light-hearted manner. Playfulness has been shown to mediate the relationship between CPTSD and relationship quality in this study, which is consistent with the findings of (Proyer 2014b) and (Brauer et al. 2021) that higher levels of playfulness in partners are associated with higher relationship satisfaction and lower conflict.

Consistent with prior research, the current findings indicate that playfulness is positively associated with trust, intimacy, and passion-core components of relationship quality. For instance, (de Moraes et al. 2021) identified playfulness as both a direct strategy for partner attraction and maintenance and an indirect process for emotional engagement through humor and flirtation. Similarly, studies using the Actor-Partner Interdependence Model (APIM) have shown that both individual and partner playfulness are associated with higher relationship satisfaction (Brauer et al., 2023b; Proyer et al., 2019).

Furthermore, the current findings align with previous studies showing the interpersonal benefits of playfulness in romantic relationships. Existing literature indicates that individuals perceive playfulness as being associated with greater partner wellbeing, more frequent mutual emotional exchange, and deeper relational bonds, a pattern linked to the maintenance of high-quality romantic relationships (Farley et al., 2021; Proyer, 2014b). These patterns were reflected in the present study, suggesting that even in the context of CPTSD, playfulness may correspond with more adaptive relationship quality. Additionally, evidence from randomized controlled trials demonstrates that structured playful activities can enhance wellbeing and reduce depressive symptoms (Proyer et al., 2021), underscoring the broader emotional significance of playfulness in adult functioning.

These findings support theoretical models suggesting that playfulness creates a “safe space” for suspending defensive states and engaging in emotionally rewarding interactions (Tuber, 2019; Winnicott, 1965, 1971). Building on (Jent et al. 2011), play represents a crucial interpersonal process associated with relationship formation, maintenance, and repair through mutual responsiveness and conflict negotiation. Playful activities such as shared laughter are linked to greater intimacy and trust (Kurtz and Algoe, 2015; Stucky, 2025), aligning with the socio-interpersonal model of trauma (Maercker and Hecker, 2016) and attachment theories emphasizing playful interactions in emotional safety (Breidenstine et al., 2011; Flynn and Falkenstien, 2024; Mikulincer and Shaver, 2019). This study underscores the resilience-related resources of mentalization and playfulness in sustaining intimate relationships among CPTSD survivors.

### 4.3 Cultural considerations

This study's findings require contextualization within Chinese cultural values. The interdependent self-construal prevalent in Chinese society suggests that disturbances in self-organization may manifest as relational concerns rather than as purely intrapsychic pathology, as negative self-appraisals in collectivist contexts often reflect perceived inadequacies in fulfilling social roles (Hu et al., 2018; Vaswani et al., 2022). Mentalization may operate through implicit, nonverbal channels in Chinese communication patterns, where tacit understanding is privileged over explicit mental state discourse (Pang et al., 2024). Similarly, playfulness operates within Confucian norms of propriety, favoring intellectual wit over exuberant emotional expression (Proyer et al., 2018). Accordingly, mentalization and playfulness may exhibit culturally variant expressions, wherein their buffering effects on relationship quality are contingent upon congruence with context-specific norms of emotional moderation and preferences for implicit communication. Furthermore, Chinese familial interdependence and Guanxi dynamics mean intimate relationships are embedded within multigenerational networks. Mentalization and playfulness may function protectively by enabling navigation of complex family obligations. These cultural mechanisms warrant investigation in future emic research to ensure interventions are culturally responsive.

### 4.4 Strengths, limitations and future directions

This study presents a comprehensive model by integrating both mentalization and playfulness as mediating factors between CPTSD and relationship quality. This dual-pathway approach underscores the roles of cognitive-emotional regulation (via mentalization) and positive affective traits (via playfulness), thereby providing a nuanced understanding of how complex trauma influences relationships. Although much of the existing literature on CPTSD focuses on individual psychopathology, this study shifts attention to the interpersonal domain, highlighting the impact of trauma on key relationship dimensions, such as intimacy, trust, and love. This interpersonal emphasis fills an important gap in the trauma literature.

The current study employs a cross-sectional design, which inherently limits causal inferences regarding the mediating effects; the observed indirect effects reflect statistical associations rather than causal processes. Consequently, the observed associations between CPTSD, mentalization, playfulness, and relationship quality may reflect reciprocal influences or third-variable confounds rather than unidirectional mediation pathways, precluding definitive conclusions regarding the temporal precedence of these constructs. Although the hypothesized model proposes directional relationships among the variables, longitudinal or experimental studies are necessary to confirm these pathways and elucidate the temporal dynamics involved. Additionally, the reliance on self-report measures to assess CPTSD symptoms, mentalization, playfulness, and relationship quality may introduce biases such as social desirability and inaccurate self-perception, as documented in the literature (Podsakoff et al., 2003). Future research should incorporate multi-method approaches, including partner reports and observational data, to enhance the validity and robustness of the findings. Furthermore, the generalizability of the findings may be constrained by the specific population and cultural context examined in this study. For instance, cultural norms surrounding playfulness and relationship dynamics can vary significantly across different societies, which may influence the observed mediating effects. The predominance of young adults in our sample limits the generalizability of findings to older populations with CPTSD.

Future research should adopt longitudinal designs to track changes in mentalization, playfulness, and relationship quality over time, thereby establishing causal pathways and identifying critical intervention periods. Experimental studies testing interventions that combine enhanced mentalization (e.g., Mentalization-Based Therapy, MBT) and increased playfulness (e.g., art or play therapy) could validate the model's practical implications and refine treatments for CPTSD (Bateman and Fonagy, 2016; Nyberg and Hertzmann, 2014; Hazar, 2019). Further research should delve into the specific symptoms of CPTSD and their intricate effects on intimate relationships, exploring additional mediating or moderating factors to achieve a comprehensive understanding of the mechanisms involved. This is essential for elucidating the multifaceted impact of CPTSD and developing targeted interventions. Cross-cultural studies should examine how mentalization and playfulness mediate the impact of CPTSD symptoms in diverse cultural contexts, potentially uncovering cultural moderators such as norms surrounding emotional expression or relational expectations. Additionally, exploring the neurobiological underpinnings of mentalization and playfulness in individuals with CPTSD could add depth to the psychological model. Identifying factors that enhance mentalization and playfulness, such as mindfulness practices, social support, or positive childhood experiences, could inform prevention and intervention strategies.

## 5 Conclusions

This study demonstrates that mentalization and playfulness function as parallel mediators in the association between CPTSD symptoms and the quality of intimate relationships. By elucidating these pathways, the findings advance our understanding of how complex trauma is associated with intimate relationship quality. Integrating approaches that enhance mentalization and foster playfulness may help trauma survivors rebuild trust, emotional connection, and adaptive relational functioning within intimate partnerships.

## Data Availability

The raw data supporting the conclusions of this article will be made available by the authors, without undue reservation.
